# Supplementary material for: The Evolution and Characterization of the RNA Interference Pathways in Lophotrochozoa
Source: Genome Biol Evol. 2024 May 7;16(5):evae098. doi: 10.1093/gbe/evae098 (PMC11114477; doi:10.1093/gbe/evae098)
Supplement: evae098_Supplementary_Data [file evae098_supplementary_data.zip › Supplementary_materials.zip!/selected_miRNAs/DaRe_selected_miRNas/DaRe_NC_007131.7_35312.pdf]

Provisional ID	:	NC_007131.7_35312
Score total	:	14.1
Score for star read(s)	:	3.9
Score for read counts	:	6.8
Score for mfe	:	2.4
Score for randfold	:	1.6
Score for cons. seed	:	-0.6
Total read count	:	25
Mature read count	:	19
Loop read count	:	0
Star read count	:	6

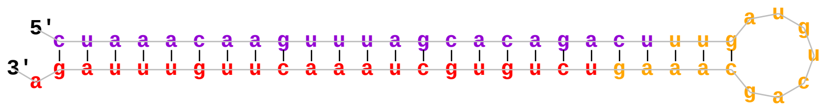

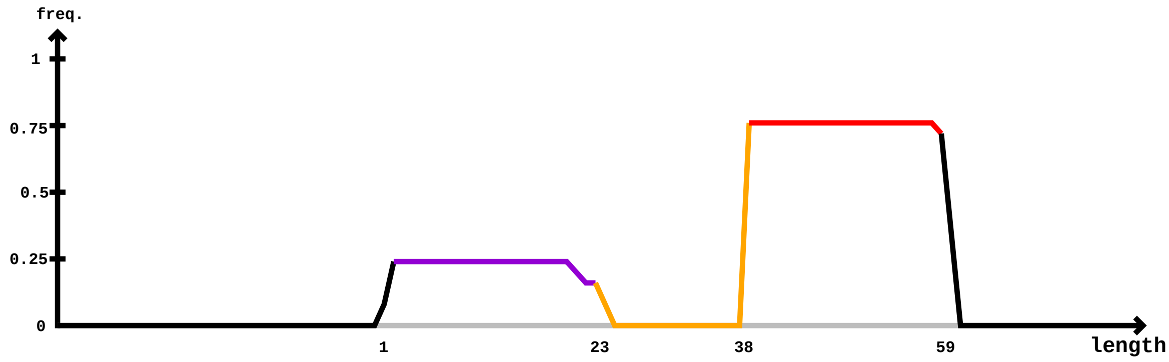

Star Mature

```
5'- auuuacgacaucaauaugaaaaaaacagcuauucuaaacaaguuuagcacagacuuugaugucagcaaagucugugcuaaacuuguuuagaauagcuguuuauuagaaugu   -3'    obs
    auuuacgacaucaauaugaaaaaaacagcuauucuaaacaaguuuagcacagacuuugaugucagcaaagucugugcuaaacuuguuuagaauagcuguuuauuagaaugu          exp
    ......................((((((((((((((((((((((((((((((((((((.......))))))))))))))))))))))))))))))))))))..........    reads      mm          sample
    ................................ucuGaacaaguuuagcacaga..........................................................    1          1           Gu3
    .................................cuGaacaaguuuagcacagacu........................................................    1          1           Gu3
    ......................................................................ucugugcuaaacuuguuuGga....................    1          1           Gu3
    ......................................................................ucugugcuaaacuuguuuaga....................    3          0           Gu3
    ......................................................................ucugugcuaaacuuguuuagaa...................    1          0           Gu3

    ......................................................................ucugugcuaaacuuguuuaga....................    1          0           Ov1

    .................................cuGaacaaguuuagcacagacu........................................................    1          1           Ov2
    ......................................................................ucugugcuGaacuuguuuaga....................    1          1           Ov2

    .................................cuGaacaaguuuagcacagacu........................................................    1          1           Gu2
    ......................................................................ucugugcuaaacuuguuuaga....................    2          0           Gu2

    ......................................................................ucugugcuGaacuuguuuaga....................    1          1           Ov3
    ......................................................................ucugugcuaaacuuguuuagaa...................    1          0           Ov3

    ................................ucuGaacaaguuuagcacag...........................................................    1          1           Gu1
    .................................cuGaacaaguuuagcacagacu........................................................    1          1           Gu1
    ......................................................................ucugugcuaaacuuguuuag.....................    1          0           Gu1
    ......................................................................ucugugcuaaacuuguuuaga....................    6          0           Gu1
    ......................................................................ucugugcuGaacuuguuuaga....................    1          1           Gu1
```
